# Successful intraoperative management in patients with abdominal compartment syndrome induced by giant liposarcomas: Two case reports

**DOI:** 10.1097/MD.0000000000022575

**Published:** 2020-10-02

**Authors:** Huaqin Liu, Tao Hu, Yuekao Li, Zhifeng Yue, Fengjiao Zhang, Jianfeng Fu

**Affiliations:** a Department of Anesthesiology; b Department of Radiology, Fourth Hospital of Hebei Medical University, Shijiazhuang, Hebei, P.R. China.

**Keywords:** abdominal compartment syndrome, intra-abdominal hypertension, liposarcoma, mechanical ventilation, transesophageal echocardiography

## Abstract

**Rationale:**

Giant intra-abdominal liposarcomas weighing over 20 kg often increase the intra-abdominal pressure (IAP), which has severe effects on the cardiovascular and respiratory systems. Abdominal compartment syndrome is defined typically as the combination of a raised IAP of 20 mm Hg or higher and new onset of organ dysfunction or failure. The anesthetic management and perioperative management are very challenging.

**Patients concerns:**

We presented 2 patients with rare giant growing liposarcoma of the abdomen, weighing 21 kg and over 35 kg, respectively. Circulatory management was particularly difficult in the first case, while respiratory management and massive blood loss was very challenging in the second one.

**Diagnosis:**

With a computed tomography scan and peritoneal-to-abdominal height ratio measurement, preoperatively the risk of developing intra-abdominal hypertension and abdominal compartment syndrome was recognized early in each patient. The inferior vena cava and right atrium of the first patient was compressed and malformed due to the uplifted diaphragm, while there was severe decreased lung volume and increased airway resistance, because of rare giant retroperitoneal liposarcomas in the second case. Histologic examination revealed dedifferentiated liposarcoma in both cases.

**Interventions:**

Both of the patients underwent resection surgery with multiple monitoring; transesophageal echocardiography monitoring in the first case and pressure-controlled ventilation volume guaranteed mechanical ventilation mode in both cases.

**Outcomes:**

Intraoperatively and postoperatively no cardiopulmonary complications in both patients. The first patient was discharged without any complications on postoperative day 10, and the second patient underwent another surgery because of anastomotic leakage resulting from bowel resection.

**Lessons:**

Multiple monitorings, in particular transesophageal echocardiography should be considered in patients with increased IAP due to a giant mass, while an appropriate lung protection ventilation strategy is crucial in these patients.

## 1 Introduction

Tumors weighing over 20 kg are considered to be giant and extremely rare.^[1]^ Giant intra-abdominal liposarcomas, especially retroperitoneal liposarcomas often increase the intra-abdominal pressure (IAP) and involve many adjacent organs and structures. A persistent increase of the IAP of ≥12 mm Hg is defined as intra-abdominal hypertension (IAH).^[2]^ IAH can cause organ dysfunction and induce the life-threatening phenomenon known as abdominal compartment syndrome (ACS), which is defined as the combination of an raised IAP of 20 mm Hg or higher and new onset of organ dysfunction or failure.[^2^,^3^] Intraoperatively, more attention should be paid to the effects of severe IAH and ACS on the cardiovascular, respiratory and urinary systems, which poses significant challenges to anesthesiologists with the American Society of Anesthesiologists standard monitors.[^4^,^5^,^6^] In addition, removal of liposarcomas from rare primary sites is also always challenging.[^7^,^8^] Herein, we would like to report our successful intraoperative management using FloTrac/Vigileo^TM^, transesophageal echocardiography (TEE) and the lung protection strategy in 2 patients with severe IAH induced by giant growing liposarcomas in the abdomen, weighing 21 kg and over 35 kg, respectively. To the best of our knowledge, this is the first time that TEE measurement and pressure-controlled ventilation volume guaranteed (PCV-VG) mode are described during the intraoperative management of a patient with giant abdominal liposarcoma.

Written informed consents were obtained from both patients for the publication of this case report.

## 2 Case reports

### 2.1 Patient 1

**Figure 1:**
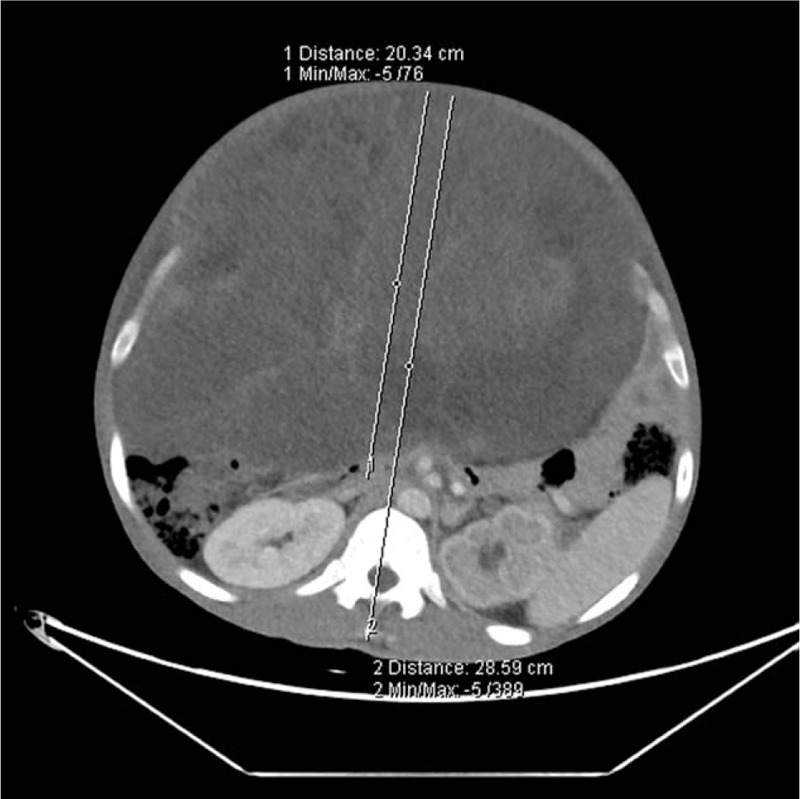
PAR (arrows) was measured as 0.71 in the first case. PAR = peritoneal-to-abdominal height ratio.

**Figure 2:**
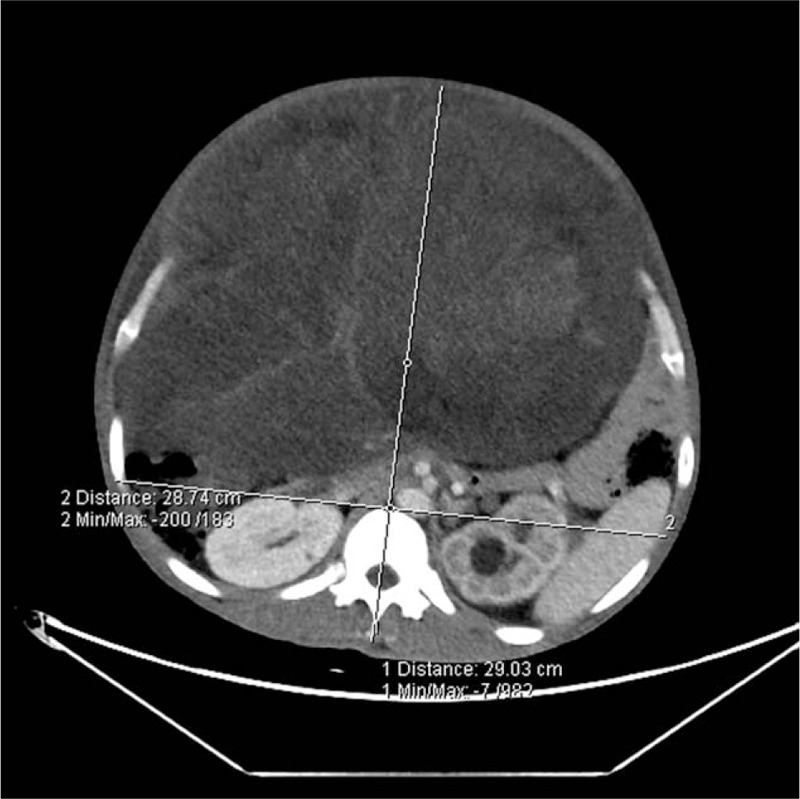
RBS (ratio of maximal anteroposterior to transverse abdominal diameter exceeding 0.8) was positive in the first case. RBS = round belly sign.

**Figure 3:**
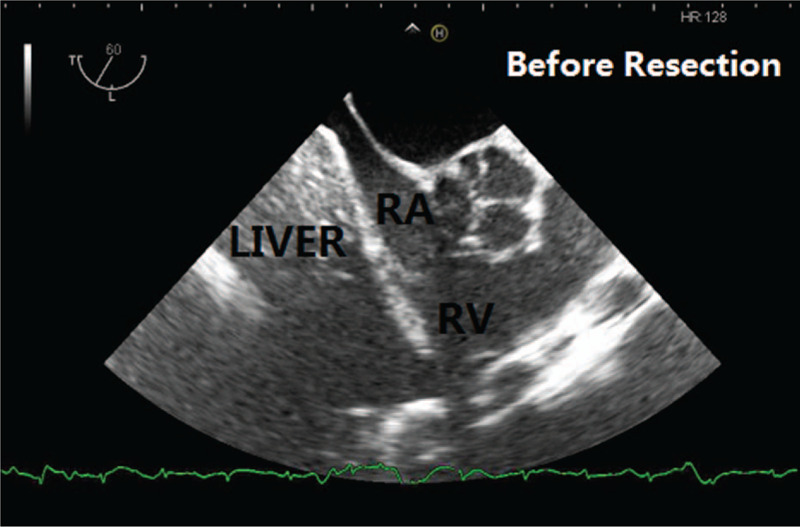
TEE image (ME RV inflow-outflow Tract) of the first patient before resection. The patient's diaphragm and liver were lifted due to compression and the right atrium's filling was limited. TEE = transesophageal echocardiography.

**Figure 4:**
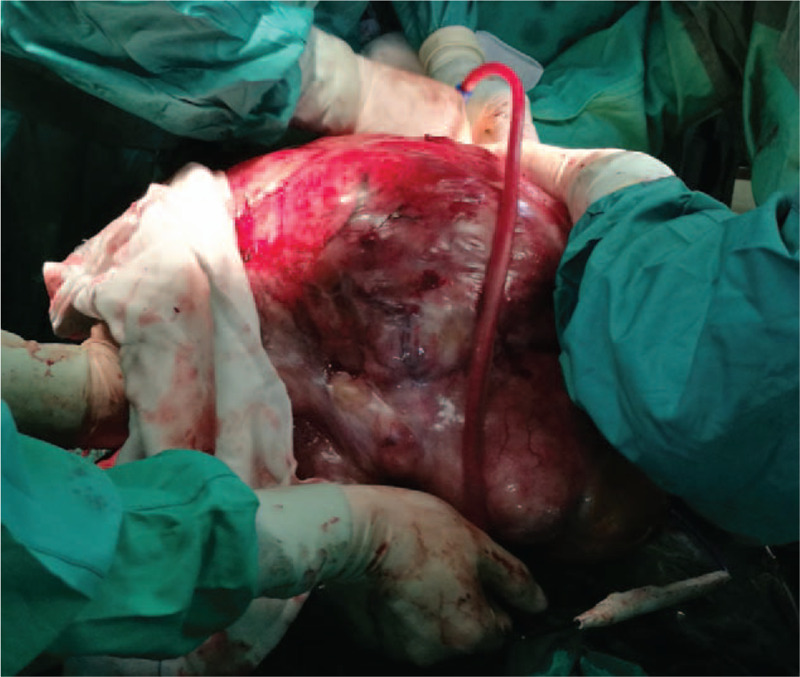
One surgical assistant was holding the tumor during the procedure.

**Figure 5:**
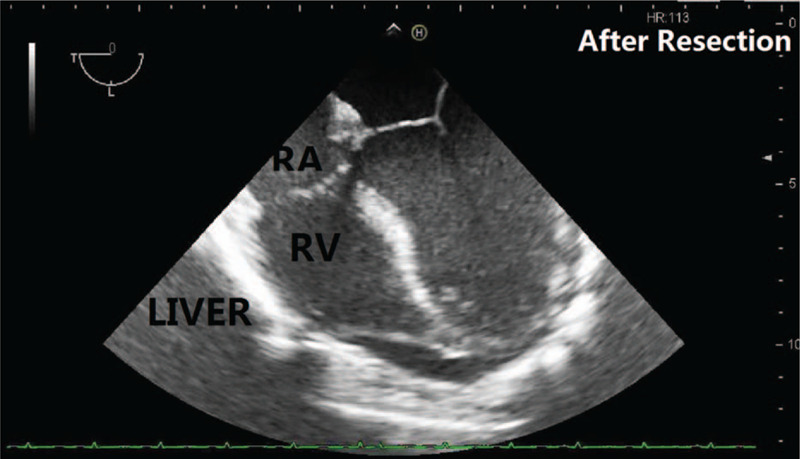
TEE image (ME 4C) of the first patient after the resection. The patient's heart returned to its normal anatomic position, and the right atrium was well filled. TEE = transesophageal echocardiography.

The first patient was a 36-year-old male (height 165 cm, weight 75 kg) presented with an abdominal liposarcoma and complaints of dyspnea and weight loss for 7 months. He refused to undergo surgery 7 months ago, because his wife was pregnant and he discontinued any further therapy. On April 15, 2019 the patient was hospitalized for an operation, since he could not adopt a supine position anymore due to shortness of breath caused by the rapid growing tumor. The abdominal computed tomography (CT) revealed a peritoneal-to-abdominal height ratio (PAR) of 0.71 (Fig. 1) and the round belly sign (defined as the ratio of maximum anteroposterior to transverse abdominal diameter exceeding 0.8) was positive (Fig. 2). The chest CT showed pulmonary atelectasis preoperatively. Laboratory tests revealed anemia and hypoproteinemia, with a progressive increase of D-Dimer. Cardiac ultrasound examination showed diastolic dysfunction and tachycardia. He was planned for resection surgery under general anesthesia combined with transversus abdominis plane block. On arrival to the operating room on April 26, 2019, the patient was put in the Semi-Fowler position. His pulse oximeter showed an oxygen saturation of 92% in room air and his heart rate was 130 to 140 beats/min. Before induction of general anesthesia, an arterial catheter was inserted into his radial artery, and arterial blood pressure, cardiac index, and stroke volume variation (SVV) were measured with a FloTract/Vigileo^TM^ system (Edwards Lifesciences, Dominican Republic). The invasive blood pressure was 150/105 mm Hg. The patient received a bilateral superior laryngeal nerve block with 2 mL of 2% lidocaine. In addition, lidocaine 2% was also sprayed on his upper airway, while dexmedetomidine 1 μg/kg was administered intravenously in 10 minutes. Heart rate fluctuated around 140 beats/min, while arterial blood pressure increased to 170/110 mm Hg. The awake fiberoptic intubation plan was immediately abandoned and slow-titration induction of general anesthesia with muscle relaxant was administered instead. Mechanical ventilation was initially volume controlled and parameters were adjusted to maintain the end-tidal carbon dioxide at 30 to 35 mm Hg, with a tidal volume (TV) of 450 to 500 mL, respiratory rates of 15, positive end-expiratory pressure (PEEP) of 5 cmH_2_O and fraction of inspired oxygen (FiO_2_) of 80% (Mindray, WATO EX-65 Pro, Shenzhen, China). As the airway pressure was 29 cmH_2_O, the ventilation mode was adjusted to PCV-VG. TEE showed a compressed and malformed inferior vena cava and right atrium (RA) due to the uplifted diaphragm (Fig. 3). A double-lumen central venous catheter (PT-01, Shenzhen Antmed Co., China) was inserted to his right internal jugular vein under ultrasound guidance and the central venous pressure (CVP) was 28 to 29 cmH_2_O. Ultrasound guided bilateral transversus abdominis plane block was administered before the surgery. After cruciate incisions were performed, the airway pressure and CVP decreased slightly. The tumor was being held by 2 surgical assistants during the whole procedure before the curative removal (Fig. 4). In the first 2 hours, CVP still remained at a higher level of 24 to 25 cmH_2_O and there was oliguria, while the SVV remained within normal range. Considering the compression of the RA and the cardiovascular effects of IAH, we increased the fluid administration rates prudently, and the urine output increased afterward. With decompression of the tumor, TEE images showed that the RA was well filled (Fig. 5) and FiO_2_ was decreased according to the results of the atrial blood gas analysis. The vital parameters remained stable while the resected specimen was removed. The tumor measured 60 cm × 50 cm × 40 cm in size and weighed 21 kg. Total surgery time was 8 hours and blood loss was about 2800 mL, therefore, 14 units of red cell concentrate, 1500 mL of fresh frozen plasma, and 20 units of platelet concentrate were transfused. Preoperative hemoglobin concentration was 74.7 g/L, while it was 99.6 g/L. After surgery, the patient was transferred to the intensive care unit (ICU) with intubation. He was extubated on postoperative day (POD) 2 and transferred to the surgery ward on POD 4. He was discharged without any complications on POD 10 and weighed 50.6 kg. The pathologic diagnosis was dedifferentiated liposarcoma.

### 2.2 Patient 2

**Figure 6:**
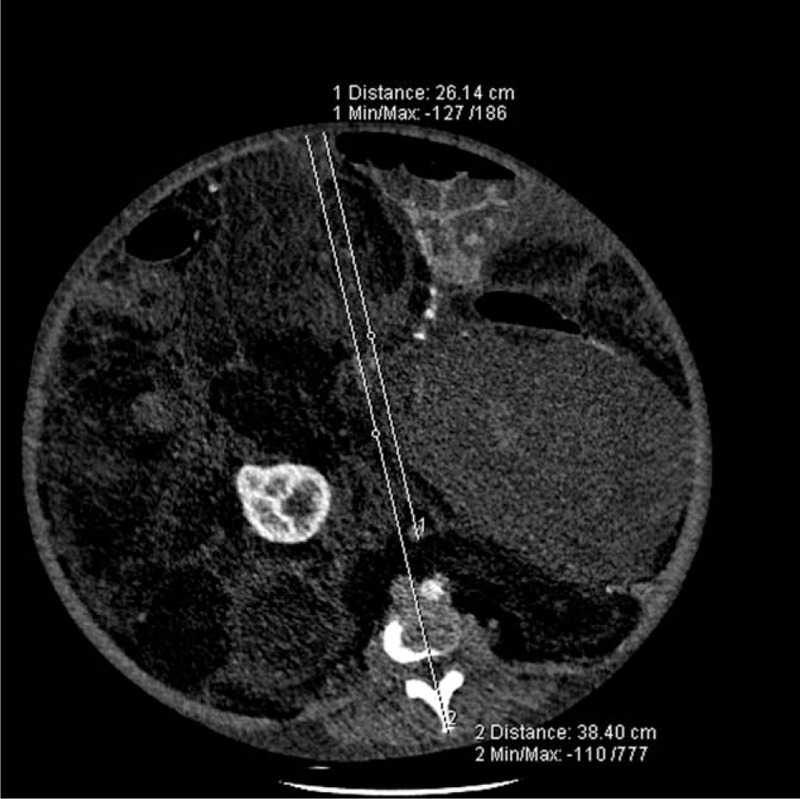
PAR (arrows) was measured as 0.68 in the second case. PAR = peritoneal-to-abdominal height ratio.

**Figure 7:**
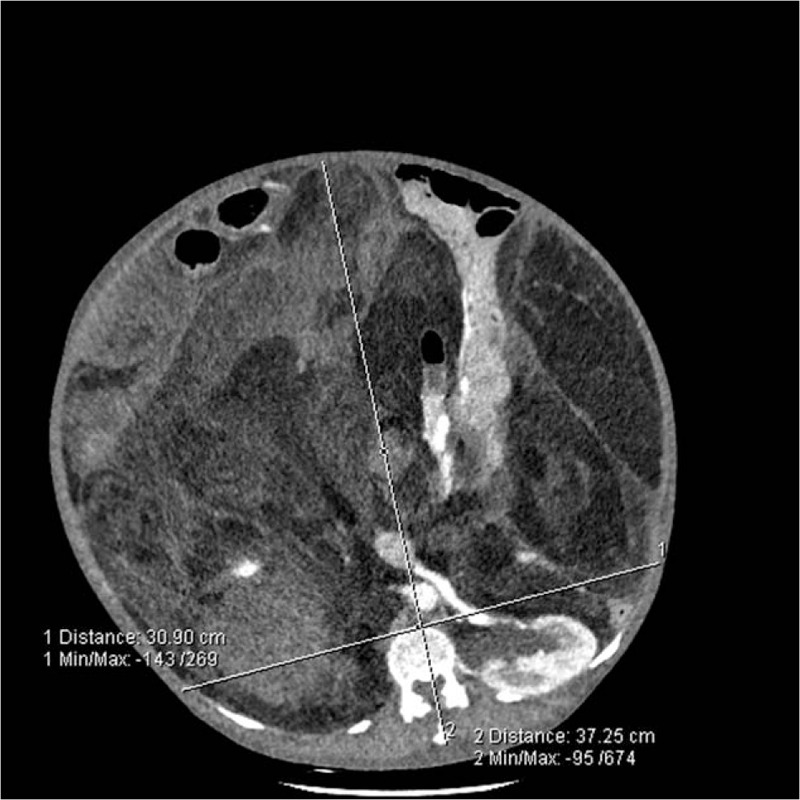
RBS (ratio of maximal anteroposterior to transverse abdominal diameter was 1.21, far exceeding 0.8) was positive in the second case. RBS = round belly sign.

**Figure 8:**
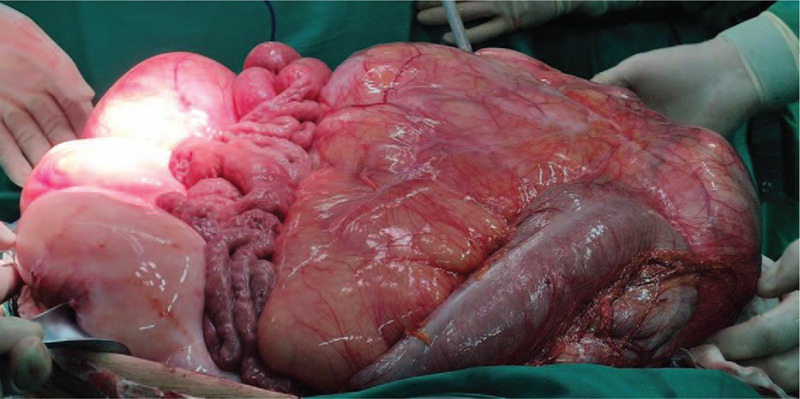
Macroscopic sandwich structure formed by the intestinal canal and mass of the second case.

**Figure 9:**
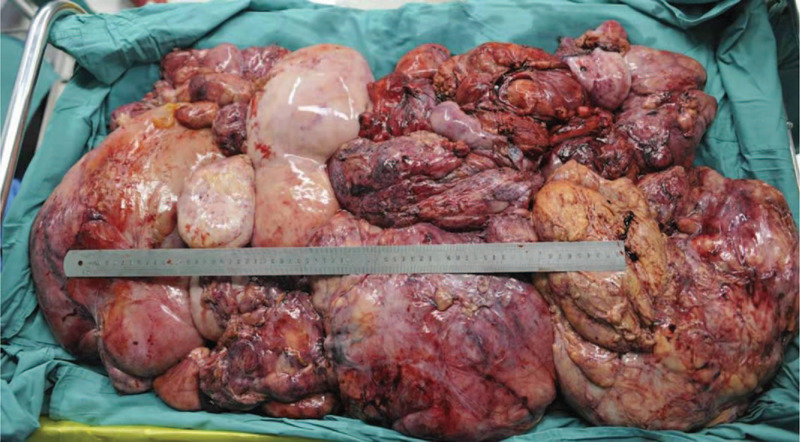
The excised surgical specimen in the second case, weighing 35 kg, which was identified on pathological examination as dedifferentiated liposarcoma.

Second case was a 53-year-old female (height 165 cm, weight 85 kg) without relevant past medical history suffering from dyspnea caused by a giant retroperitoneal liposarcomas that progressed over 2 years after invalid conservative treatment. A hard mass occupying the entire abdomen was palpated on physical examination and the patient had cachexia. Abdominal CT revealed a PAR of 0.68 (Fig. 6) and the ratio of maximum anteroposterior to transverse abdominal diameter was 1.21 (Fig. 7). Preoperative biopsy showed a mesenchymal tumor. Laboratory tests showed anemia and severe hypoproteinemia. Left chamber enlargement and impaired left ventricular relaxation were showed by echocardiography. The patient was planned for en bloc resection surgery under general anesthesia without TAP block on August 12, 2019. Arterial blood gas analysis showed arterial partial pressure of carbon dioxide of 40.8 mm Hg and arterial partial pressure of oxygen of 50.0 mm Hg in room air. With the FloTract/Vigileo^TM^ system, the invasive blood pressure was 150/80 mm Hg. In consideration of the stable hemodynamic parameters and our experience from the previous case, the same general anesthesia protocol was implemented as described in the first case. After the anesthesia induction, the patient was ventilated in a volume-controlled mode. As the tumor weight was estimated around 40 kg, the TV was set to 450 mL, with respiratory rate of 16 beats/min, PEEP of 5 cmH_2_O and airway pressure of 30 cmH_2_O. A FiO2 of 0.8 showed a PaCO2 of 44.7 mm Hg, PaO2 of 68.9 mm Hg and the CVP was measured as 25 cmH_2_O. During surgery, a midline xyphoid to pubic incision was performed, and the airway pressure decreased gradually therewith. To obtain a sufficient arterial oxygenation, we couldn’t decrease the FiO_2_ until the tumor was excised partly 3 hours later. A typical sandwich structure was formed by the intestinal canal and the mass (Fig. 8). As the tumor encased the entire right kidney and had invaded the small intestine, a right nephrectomy and combined resection was performed, and there was massive blood loss during the whole surgery. Her total blood loss was 12800 mL and 64 units of red cell concentrate, 4800 mL of fresh frozen plasma, 40 units of cryoprecipitate and 3 units of platelet concentrate were transfused. Preoperative hemoglobin concentration was 106 g/L after blood transfusion, while it was only 82.0 g/L. The tumor was removed mainly, weighed 35 kg (Fig. 9), and was identified by pathological examination as dedifferentiated liposarcoma. On completion of the surgery she was transferred to the ICU with intubation. She was extubated on POD 3 and discharged from the ICU on POD 6. She underwent another surgery, because of anastomotic leakage resulting from bowel resection on POD 10.

## 3 Discussion

Intraoperative management in patients with IAH/ACS induced by giant liposarcomas pose significant challenges to the anesthesiologists. As clinical examination is not accurate to detect IAH,^[9]^ it has been recommended since 2013 to estimate IAP by a standardized trans-bladder measurement, in case of any known risk factor for IAH/ACS in a critically ill or injured patient.^[2]^ However, IAP measurement is not always possible (e.g. patients presenting bladder troubles), so early recognition and treatment of ACS is a key to improving the outcome of patients. Bouveresse S., et al reported a simple, early, and relevant warning sign related to the risk of IAH and ACS; CT scan of PAR measurement.^[10]^ In our 2 cases, PAR was 0.71 and 0.68 respectively, while round belly sign were both positive, who presented with obvious dyspnea, and higher levels of CVP. Therefore, during our intraoperative management we paid more attention to features of ACS. Within the first 2 hours of surgery, both cases presented with oliguria and a higher level of CVP at normal fluid infusion rates, and the first case also developed tachycardia. We increased the fluid administration rate according to the principle of ACS, instead of cardiac insufficiency. As a result, the urine output increased while the CVP decreased in both cases, which further supported our initial judgements.

Multiple hemodynamic monitoring is of greater importance in patients with IAH induced by giant intra-abdominal liposarcoma. The CVP monitoring could not accurately reflect the right ventricular volume, because of the increased IAP.^[11]^ Monitoring of the CO, SVV, and even TEE measurements are very important during this kind of surgery. In the first case, TEE measurement was significantly useful and helpful for cardiac assessment and advanced fluid therapy.

For patients with ACS, increased IAP leads to decreased lung volume and compliance, and increased airway resistance, which results in hypoxemia and even acute respiratory distress syndrome. Up to now, there is no appropriate ventilation mode recommended for patients with ACS due to giant intra-abdominal liposarcoma, who require surgery under general anesthesia. As for the patients with ACS requiring mechanical ventilation support in the ICU, pressure-regulated volume control ventilation was used as a lung protection strategy.^[12]^ Compared with pressure control ventilation, pressure-regulated volume control ventilation provides satisfactory and stable ventilation at the lowest possible pressure level, which relieves injury by positive pressure ventilation and increases safety. PCV-VG is a mechanical ventilation mode that guarantees TV with the lowest possible pressure using decelerating flow for patients undergoing surgery.[^13^,^14^,^15^] As for our 2 cases, intraoperative high airway pressures and barotrauma were also of great concern, therefore, PCV-VG ventilation mode was adjusted for each patient. It was also reported that spontaneous ventilation should be maintained for as long as possible and that the inspiratory pressure should be kept under 20 cmH_2_0 even if muscle relaxants were used.^[2]^ Although in our experience, as for the solid giant liposarcoma, anesthesia induction with muscle relaxant could reduce the respiratory system compliance greatly. The airway pressures in both cases were up to almost 30 cmH_2_0 even with the muscle relaxants, while it decreased significantly following abdominal resection of the tumor. Thus shortening the interval time between the anesthesia induction and operation initiation was very important. Previous reports also stated that there was a risk of re-expansion pulmonary edema (RPE) after removal of the tumor.^[16]^ RPE develops upon rapid expansion of chronically collapsed lungs through the mechanism of increased pulmonary vascular permeability and there is no standard method to prevent it. The first case presented with pulmonary atelectasis preoperatively, while the second case did not. RPE did not occur in both cases. Using the low-tidal-volume with PEEP practice and PCV-VG ventilation mode, we were able to manage the patients during the operation and postoperatively without respiratory complications.

Growing tumors are usually hypervascular, which poses great risks of severe bleeding.^[2]^ The supplying vessels of the tumor can be distinguished with selective angiography preoperatively and ligating them could greatly reduce blood loss during operation. The abdominal circumferences of both patients increased progressively during their hospitalization due to the growing tumors. Unfortunately, our both cases did not undergo preoperative ligation due to technical difficulties.

Intraoperative hypothermia in giant intra-abdominal liposarcoma patients should be avoided, since there is exposure of peritoneal cavity for prolonged operation times and transfusion of large volumes of intravenous blood products and fluid. We kept the patients actively warm using a circulation water blanket and forced-air warming blanket.

In conclusion, IAH and ACS are increasingly recognized as risk factors for organ failure and mortality among a wide variety of patient populations.^[17]^ More attention should be paid to the intraoperative effects of severe IAH and ACS. Multiple monitorings, in particular TEE should be considered in patients with increased IAP due to a giant mass, while an appropriate lung protection ventilation strategy is crucial in these patients.

## Author contributions

**Data curation:** Yuekao Li.

**Investigation:** Tao Hu, Zhifeng Yue, Fengjiao Zhang.

**Resources:** Zhifeng Yue, Fengjiao Zhang.

**Writing – original draft:** Huaqin Liu.

**Writing – review & editing:** Huaqin Liu, Jianfeng Fu.
